# Abnormal development of zebrafish after knockout and knockdown of ribosomal protein L10a

**DOI:** 10.1038/s41598-019-54544-w

**Published:** 2019-12-02

**Authors:** Kunwadee Palasin, Tamayo Uechi, Maki Yoshihama, Naparee Srisowanna, Narantsog Choijookhuu, Yoshitaka Hishikawa, Naoya Kenmochi, Wilaiwan Chotigeat

**Affiliations:** 1 Department of Molecular Biotechnology and Bioinformatics, Faculty of Science, Prince of Songkla University, Hatyai, Songkhla, 90112 Thailand; 2 Center for Genomics and Bioinformatics Research, Faculty of Science, Prince of Songkla University, Hatyai, Songkhla, 90112 Thailand; 3 Frontier Science Research Centre, University of Miyazaki, Kiyotake, Miyazaki, 889-1692 Japan; 4 Department of Anatomy, Histochemistry and Cell Biology, Faculty of Medicine, University of Miyazaki, Kiyotake, Miyazaki, 889-1692 Japan

**Keywords:** Embryogenesis, Gene expression

## Abstract

In this study, to investigate the secondary function of Rpl10a in zebrafish development, morpholino antisense oligonucleotides (MOs) were used to knock down the zebrafish ribosomal protein L10a (*rpl10a*). At 25 hpf (hours post-fertilization), embryos injected with the *rpl10a* MO showed an abnormal morphology, including short bodies, curved tails, and small yolk sac extensions. We observed pigment reductions, edema, larger yolk sacs, smaller eyes and smaller yolk sac extensions at 50 hpf. In addition, reductions in the expression of primordial germ cell (PGC) marker genes (*nanos1* and *vasa*) were observed in *rpl10a* knockdown embryos. A rescue experiment using a *rpl10a* mRNA co-injection showed the recovery of the morphology and red blood cell production similar to wild-type. Moreover, the CRISPR-Cas9 system was used to edit the sequence of *rpl10a* exon 5, resulting in a homozygous 5-bp deletion in the zebrafish genome. The mutant embryos displayed a morphology similar to that of the knockdown animals. Furthermore, the loss of *rpl10a* function led to reduced expression of *gata1*, *hbae3*, and *hbbe1* (erythroid synthesis) and increased *tp53* expression. Overall, the results suggested that Rpl10a deficiency caused delays in embryonic development, as well as apoptosis and anemia, in zebrafish.

## Introduction

Ribosomal proteins (RPs) function in protein synthesis. Many lines of evidence suggest other roles for RPs, including DNA repair, apoptosis, translational regulation, developmental control, and red blood cell development^1^. Several RP mutations are associated with Diamond-Blackfan anemia (DBA) patients and include *RPS19*, *RPS7*, *RPS10*, *RPS17*, *RPS24*, *RPS26*, *RPL5*, *RPL11*, *RPL26*, and *RPL35A*^2–9^. The mutation of some RPs (e.g., RPL5, RPL11, RPL23) may cause a failure in ribosome biogenesis and the accumulation of free RPs that can trigger Tp53-mediated apoptosis; defects in ribosome biogenesis cause aberrations in red blood cells and thereby activate *tp53* in the erythroid lineage^10^. Thus, proper ribosome formation is a checkpoint for cell cycle progression^11^. Additionally, *rpl11* mutation or loss of Rpl11 function activates a Tp53-dependent checkpoint response to prevent abnormal embryonic development^12^.

Ribosomal protein L10a (Rpl10a) is in the L1P family of ribosomal proteins and is encoded by the *rpl10a* gene. A previous study showed that the Rpl10a protein might play an important role during embryogenesis and organogenesis^13^. Recombinant Rpl10a protein was also shown to be involved in shrimp ovary development both *in vitro* and *in vivo*^14,15^. Although Rpl10a has secondary functions in embryogenesis and ovary development, the effects of Rpl10a on primordial germ cells, or PGCs, during early embryogenesis have not been reported. PGCs are the precursors to reproductive gametes (spermatozoa and oocytes). The *vasa* gene has been identified as a marker of PGCs in zebrafish^16^, and *nanos1*, a germplasm gene, is essential for PGC migration and viability^17^. In *Drosophila*, PGCs deficient in *Nanos* activity showed abnormal development, shifts to gonad failure, a reduction in egg number, and morphological abnormalities^18^. These results in fruit flies were similar to results from zebrafish^17^.

Therefore, we knocked down and knocked out the *rpl10a* gene using morpholinos and CRISPR-Cas9, respectively, to investigate the function of the *rpl10a* gene in embryogenesis, germ cell development, and erythropoiesis. Quantitative RT-PCR was performed to determine the fold changes in *gata1* and *tp53* gene expression in *rpl10a-*deficient embryos. The expression of the zebrafish embryonic globin genes, hemoglobin alpha embryonic-3 (*hbae3*) and hemoglobin beta embryonic-1 (*hbbe1*), which were highly expressed at 48 hpf^19^, were used as markers of erythroid cells. Moreover, histology was used to support the morphological defects and the effects of *rpl10a* knockdown on PGC marker genes, including *nanos1* and *vasa* gene expression, which were investigated using qRT-PCR and whole-mount *in situ* hybridization.

## Results

### Zebrafish *rpl10a* knockdown and knockout construction

**Figure 1:**
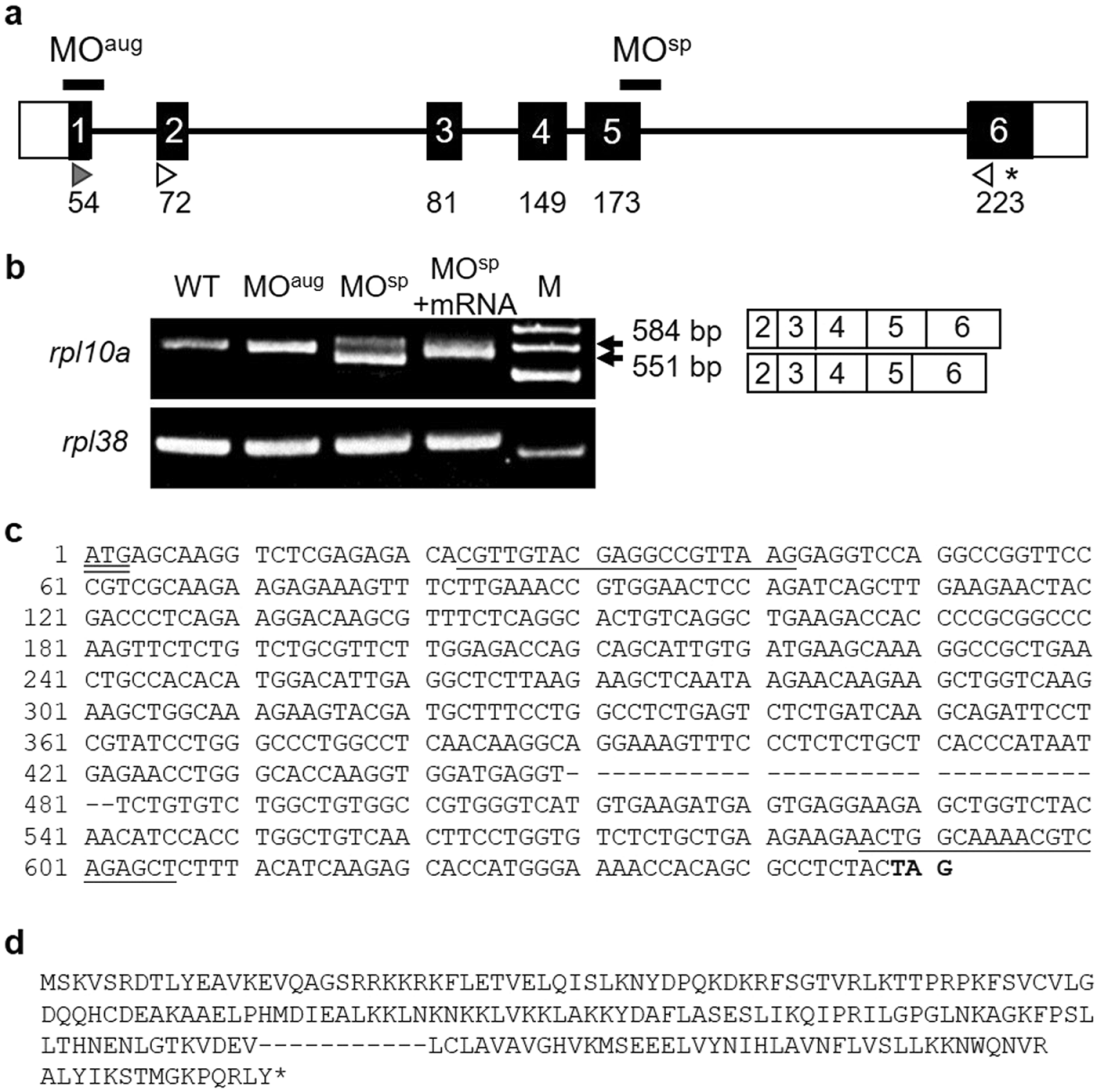
(**a**) Schematic of the zebrafish *rpl10a* gene structure. White boxes represent the untranslated regions, while the translated region is shown with black boxes. The start codon (gray arrowhead) and stop codon (asterisk) positions are presented. The white arrowheads are marked at the position of the MO^sp^ detection primer, and the arrowheads indicate the direction of PCR polymerization. The positions of the designed MO^aug^ and MO^sp^ are indicated. (**b**) RT-PCR analysis of *rpl10a* in MO-injected (MO^aug^, MO^sp^), rescued (MO^sp^ + mRNA) and wild-type embryos; *rpl38* was amplified as a control. M was shown as a 100 bp ladder. A smaller PCR product (551 bp) was observed from MO^sp^-injected embryos because 33 bp of exon 5 was skipped; the wild-type product was 584 bp (primers were designed to obtain products smaller than the full-length gene). (**c**) The nucleotide sequences of *rpl10a* cDNA are presented. The hyphens displayed 33 bps of exon 5 that were deleted after MO^sp^ injection. The underline shows the position of primer sequences. The start codon and stop codon appeared in double underline and bold text, respectively. (**d**) The amino acids were predicted using the translation tool from www. ExPASy.com. The 11 amino acids were predicted to be removed after deletion. Hyphens showed deleted amino acids, and the stop codon is shown in an asterisk.

The zebrafish *rpl10a* gene is located on chromosome 8 and contains six exons, resulting in a 648 bp cDNA sequence. The *rpl10a* gene encodes a protein of up to 216 amino acids. To investigate the effect of Rpl10a deficiency, we knocked down the *rpl10a* gene using MO injection to inhibit protein translation. The MO target sites are shown in the diagram in Fig. 1a. The injection of MO^sp^, with specificity to exon 5, altered the splicing and resulted in the exclusion of some of exon 5 from the mature mRNA. Moreover, the expression of the *rpl10a* gene decreased, as detected by RT-PCR (Fig. 1b). DNA sequencing confirmed the deletion in the splice site of exon 5 after MO^sp^ injection. We found that only 33 bps of exon 5 were deleted (Fig. 1c), and 11 amino acids were predicted to be deleted (Fig. 1d).

We used the CRISPR-Cas9 genomic editing system using the crRNA-tracrRNA-Cas9 complex to edit exon 5 of the *rpl10a* gene in zebrafish. From the domain prediction using ScanProsite, we found that the Rpl10a protein contains 1 domain, namely, the *ribosomal protein L1* signature. This domain displays RNA chaperone activity with the amino acid sequence IKQIPRILGPGLNKAGKFPS, and these sequences were encoded by exon 5.

**Figure 2:**
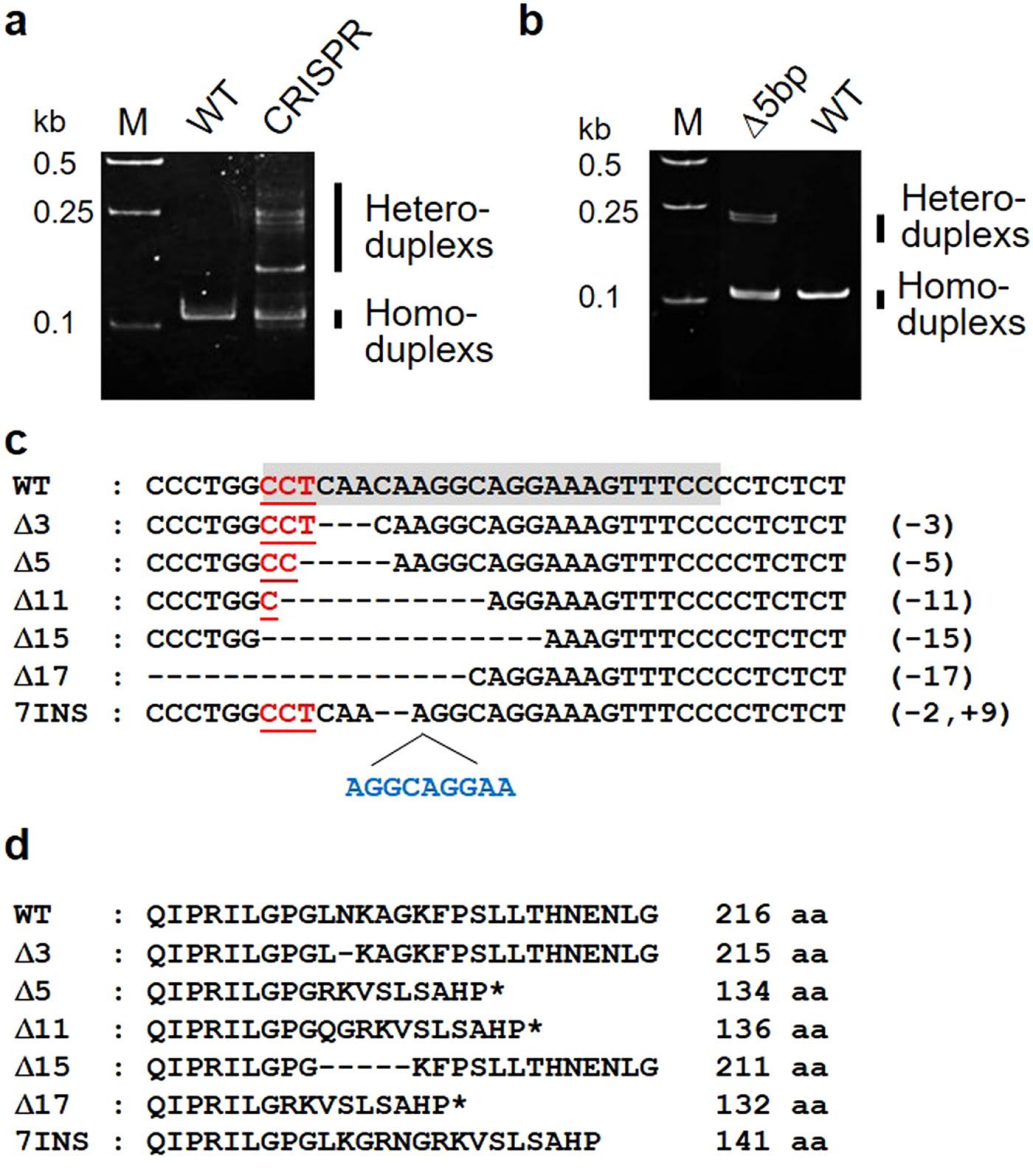
(**a**) Pattern of heteroduplexes and homoduplexes observed after crRNA-tracrRNA-Cas9 complex (CRISPR-Cas9) injection. (**b**) HMA showing different heteroduplex and homoduplex patterns in the 5-bp deletion mutant compared to the wild-type (WT). (**c**) Schematics of the mutations obtained from genome editing. The crRNA target site and PAM sequences are presented with gray highlighting and red letters, respectively. Deleted nucleotides are shown with hyphens, and inserted nucleotides are in blue letters. (**d**) Predicted amino acid sequences after *rpl10a* gene knockout by CRISPR-Cas9 starting from amino acid position 190. The deleted sequences and stop codons are marked with hyphens and stars, respectively.

Additionally, the heteroduplexes of F0 mutant fish were detected via heteroduplex mobility assay (HMA) after CRISPR-Cas9 injection (Fig. 2a). The 115 embryos slowly died off after injection and only 18 became adult. They were screened for mutations transmitted through the germline. We selected progeny with positive germline transmission based on their patterns. The HMA results of the 5-bp deletion mutants showed a different pattern of heteroduplexes and homoduplexes in the heterozygous mutant (Fig. 2b). The heteroduplexes migrated more slowly than the homoduplexes due to structural distortion. The mutant genotype was confirmed by sequencing (Fig. 2c). Among the 18, eight mutated F0 were found to transmit a lesion into their F1 progenies, 6 adults were wild-type and the other 4 could not give F1 progeny. The number of F1 founders obtained from the F0 generation was 40 fishes. The F1 progenies had 6 mutation patterns, including a 3-bp deletion, a 5-bp deletion, an 11-bp deletion, a 15-bp deletion, a 17-bp deletion, and a 7-bp insertion. Amino acid changes were predicted based on the gene mutations (Fig. 2d). These patterns corresponded to 215, 134, 136, 121, 132 and 141 amino acids in the order mentioned above in F1 generation fish. For further analysis, the 5-bp deletion was used as a model to study the loss of the function of *rpl10a* gene.

### Morphological defects in *rpl10a* dysfunction

**Figure 3:**
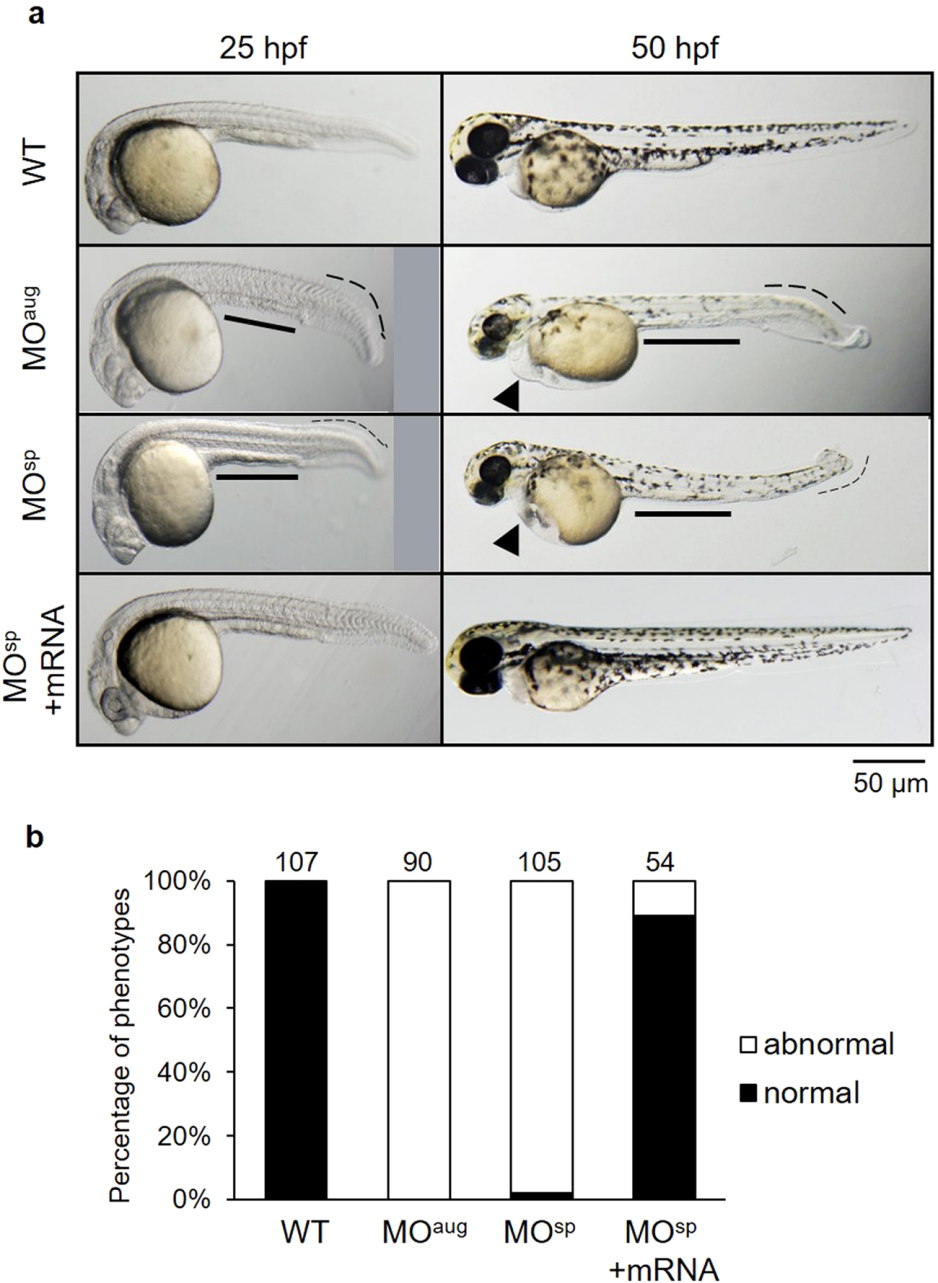
(**a**) Effect of Rpl10a deficiency on embryonic development. Lateral views of *rpl10a* MO-injected embryos and rescue phenotype observed at 25 and 50 hpf. At 25 and 50 hpf, smaller eyes, yolk sac extensions, bent tails, and developmental delays were observed in *rpl10a* knockdown. At 50 hpf, deficient embryos displayed smaller eyes with reduced pigmentation and edema (arrowheads). The MO^sp^ + mRNA injected embryos displayed rescue phenotypes. (**b**) The phenotype of *rpl10a* MO^aug^ (n = 90), MO^sp^ (n = 105), and MO^sp^ + mRNA (n = 54) injected embryos was evaluated compared to wild-type (n = 107) at 25 hpf. The morphologic abnormalities include a thinner yolk sac extension, a shorter body length, and a bent tail, whereas the normal wild-type embryos showed a normal yolk sac extension and a straight tail. The result was presented in the percentage of normal (black bar) and abnormal (white bar) embryos in each group.

The phenotype of the knockdown embryos (morphants) was compared to that of wild-type embryos at approximately 25 hpf. The *rpl10a* MO-injected embryos showed morphological abnormalities, including shorter body lengths, bent tails, and smaller yolk sac extensions. We observed a reduction in pigmentation, edema, larger yolk sacs, smaller eyes, and smaller yolk sac extensions at 50 hpf (Fig. 3a). Most *rpl10a* morphants died by 3–7 days post-fertilization (dpf). The non-injected embryos had no phenotypic changes. Rescued embryos using *rpl10a* mRNA co-injection showed morphologies similar to that of the wild-type. The phenotype in each group was evaluated and calculated as percentages of normal and abnormal phenotypes (Fig. 3b). All MO-injected embryos had a similar phenotype. However, some of them showed a moderate phenotype in the MO-injected group. In the case of MO^sp^ + mRNA, injected embryos showed a recovery phenotype of approximately 89%.

**Figure 4:**
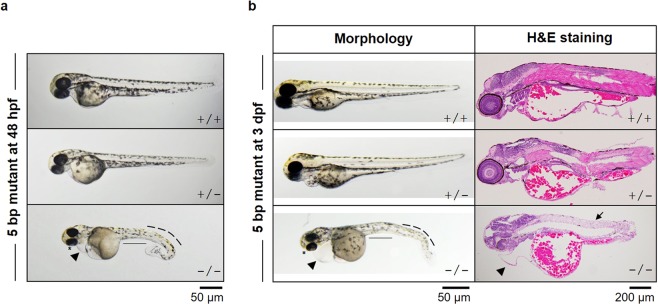
(**a**) Phenotypes of a 5-bp homozygous deletion (−/−) embryo, heterozygous deletion (+/−) and wild-type (+/+) embryos at 48 hpf are presented. Homozygous mutant embryos displayed severe phenotypes, including smaller eyes, heart and yolk sac edema, smaller yolk extension, curved tail and depigmentation. (**b**) At 3 dpf, the altered phenotypes and H&E staining were also performed.

The 5-bp deletion embryos were also used as a model to confirm the effect of Rpl10a deficiency on early embryogenesis. Embryos carrying a homozygous 5-bp *rpl10a* mutation showed a severe phenotype, and all mutant embryos died at 3–5 dpf. The phenotypes of homozygotes were more severe when compared with MO-injected embryos at 25 hpf. The heterozygous phenotype was difficult to distinguish; however, few embryos slightly differed from wild-type. Additionally, the homozygous 5 bp deletion at 48 hpf was more severe, including the curved tail, enlarged yolk sac, heart edema, depigmentation, and smaller eyes (Fig. 4a). The homozygous mutant fish at 3 dpf displayed smaller eyes, loss of pigmentation, bent tails, and edema. These morphologies were abnormal due to developmental interruption, leading to death. In addition, the *rpl10a* defect led to swelling of the yolk sac. This phenomenon indicated that nutrients in yolk sac accumulation and retaining. To confirm this without metabolic assessments of embryos, we performed H&E to stain the fluid in the yolk sac (Fig. 4b). Eosin stains the extracellular matrix and cytoplasmic parts. It is an acidic dye that binds to proteins and lipids in the cytoplasm. In this result, the pink spots in the yolk sac and yolk extension were proteins, lipids, and nutrients. The results indicated an accumulation of nutrients in the yolk sac due to low metabolism in the mutant fish. Moreover, the eosinophilic cytoplasmic filaments in muscle cells were decreased (Fig. 4b).

### Reduction in hemoglobin level with *rpl10a* defects

**Figure 5:**
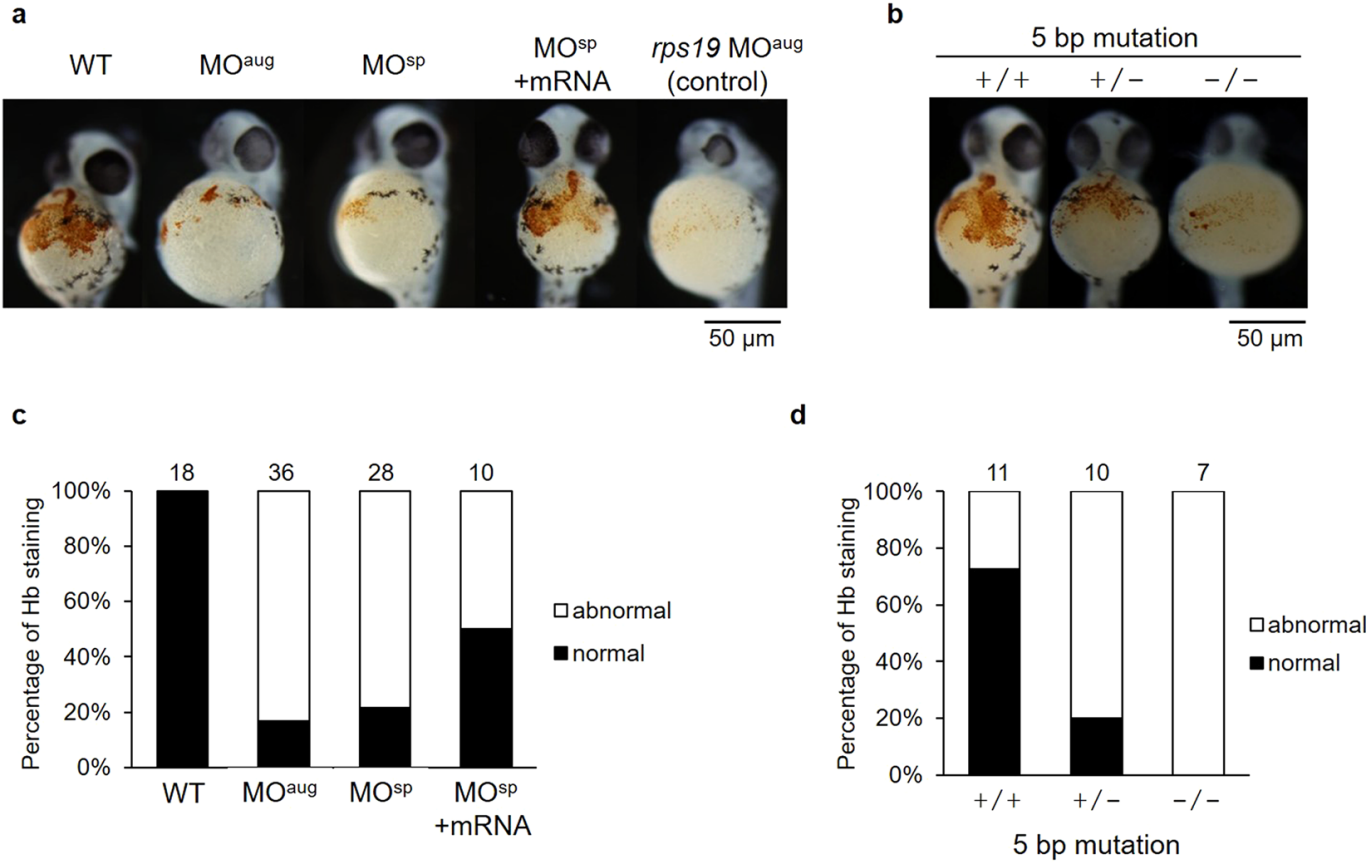
(**a**) Significant reductions in hemoglobin (Hb) staining in the embryos were observed after *rpl10a* gene knockdown (MO^aug^ and MO^sp^), similar to *rps19* knockdown, compared to the control; embryos co-injected with *rpl10a* transcript recovered at 48 hpf. (**b**) *rpl10a* embryos mutated using CRISPR-Cas9 knockout showed significant reductions in hemoglobin staining. (+/+: wild-type, +/−: heterozygous, −/−: homozygous mutant). The graph displayed the percentages of normal and abnormal levels of Hb staining in (**c**) knocked down *rpl10a* gene and mRNA rescue embryos; (**d**) 5 bp *rpl10a* mutant embryos. The dense Hb staining (orange dot) at the wild-type embryo yolk sac was considered normal while a significantly pale orange or slightly colored Hb staining in the yolk sac was evaluated as abnormal. The number of animals quantified in each group are shown on top of the bars.

Typically, the loss of ribosomal function leads to anemia in patients. We investigated the effects of *rpl10a* defects on reducing hemoglobin level at 48 hpf. Low *o-*dianisidine staining signals were detected in the *rpl10a* knockdown. The embryos rescued by mRNA co-injection showed increased staining similar to that of the wild-type (Fig. 5a). Additionally, the 5-bp *rpl10a* deletion mutants showed results corresponding to those of the knockdown. The heterozygous mutants showed reduced hemoglobin staining. A reduction in hemoglobin content was observed in the homozygous mutants (Fig. 5b). As shown in Fig. 5c, the normal hemoglobin staining level was decreased to 22% in *rpl10a* knockdown. After rescue using *rpl10a* mRNA co-injection, the number of hemoglobin recovered and showed a normal level of 50%. In addition, the reduction of hemoglobin level in homozygous mutant embryos showed more severe than heterozygotes and knockdown embryos.

### Effect of *rpl10a* defects on expression of erythropoiesis-related genes

**Figure 6:**
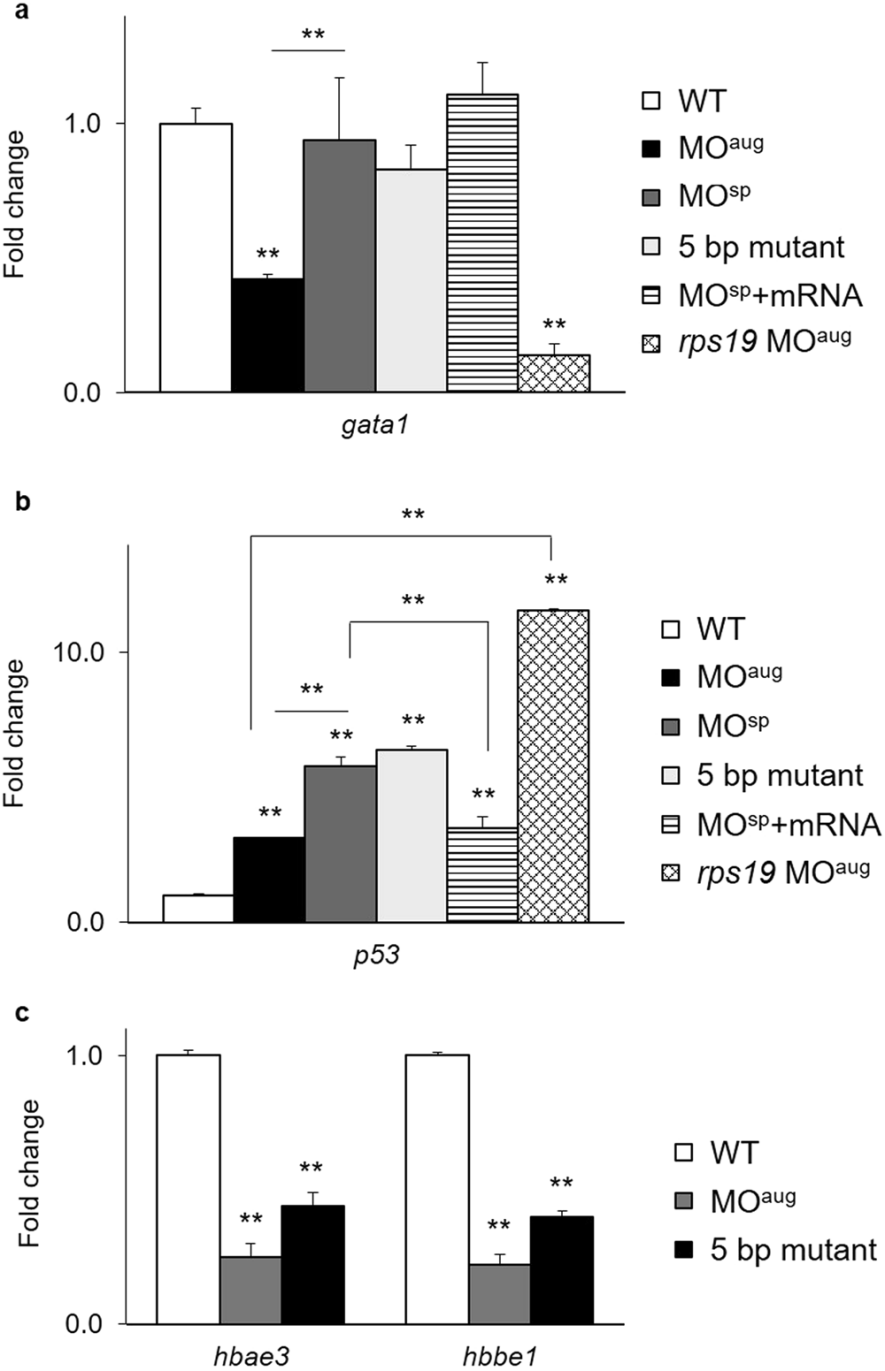
Quantitative RT-PCR results showing the fold changes in the expression of the (**a**) *gata1* and (**b**) *tp53* genes in *rpl10a* MO^aug^, MO^sp^, homozygous mutant, and MO^sp^ + mRNA injected embryos at 24 hpf compared to wild-type and *rps19* knockdown embryos as controls. (**c**) Fold changes in the expression of *hbae3* and *hbbe1* mRNA in Rpl10a-deficient embryos and mutant samples at 48 hpf. Wild-type embryos were used as controls. Each group used 20 pooled embryos (replicates = 4). The data were analyzed for statistical significance by one-way ANOVA followed by Tukey’s multiple comparison test. (**p-value > 0.01).

The fold change in the expression of the *gata1* gene, which is involved in blood cell maturation, was reduced in MO-injected embryos at 24 hpf (Fig. 6a). Additionally, *gata1* expression was downregulated in mutant embryos. However, MO^sp^-injected embryos and homozygous mutant fish showed no significant reduction. Higher expression of the *tp53* gene was detected in all *rpl10a-*deficient embryos and homozygous mutants than in the wild-type embryos (Fig. 6b). The trend of these expression results was similar to that with *rps19* MO^aug^ injection. In addition, the fold change in *tp53* expression levels declined after rescue with *rpl10a* mRNA. To check the expression of erythroid markers at the embryonic stage, *hbae3* and *hbbe1* were analyzed in the *rpl10a* knockdown and knockout embryos (Fig. 6c). The qPCR results showed that these genes, which are involved in erythroid synthesis, had low expression in Rpl10a-deficient embryos, suggesting that it might play a role in anemia.

### Reduced expression of PGC marker genes in *rpl10a*-defective embryos

**Figure 7:**
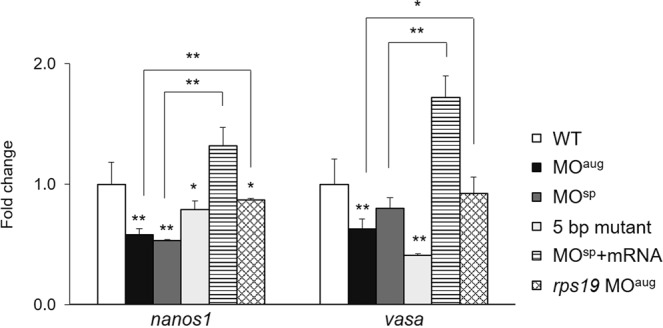
Fold change in the expression levels of *nanos1* and *vasa* in *rpl10a* MO^aug^, MO^sp^, homozygous mutant and MO^sp^ + mRNA injected embryos compared to WT and *rps19*-deficient embryos as controls. Each group used 20 pooled embryos (replicates = 4). The data were analyzed for statistical significance by one-way ANOVA followed by Tukey’s multiple comparison test. (*p-value > 0.05, **p-value > 0.01).

The fold changes in the expression of PGC marker genes after knocking down the *rpl10a* gene are presented compared to WT and *rps19* knockdown at 25 hpf. The expression levels of *nanos1* and *vasa* transcripts were reduced considerably in *rpl10a*-MO^aug^ embryos by 0.42- and 0.37-fold from WT, respectively. The relative expression levels of *nanos1* and *vasa* also significantly decreased compared to those associated with Rps19 deficiency. However, the expression of *nanos1* in Rps19*-*deficient embryos differed slightly from that of the WT. The homozygous mutant embryos showed significant decreases in *vasa* and *nanos1* mRNA expression levels. Furthermore, the *vasa* and *nanos1* transcription levels were upregulated when rescued with *rpl10a* mRNA injection (Fig. 7). The qPCR results revealed that the PGC marker genes were reduced when the *rpl10a* gene was defective.

**Figure 8:**
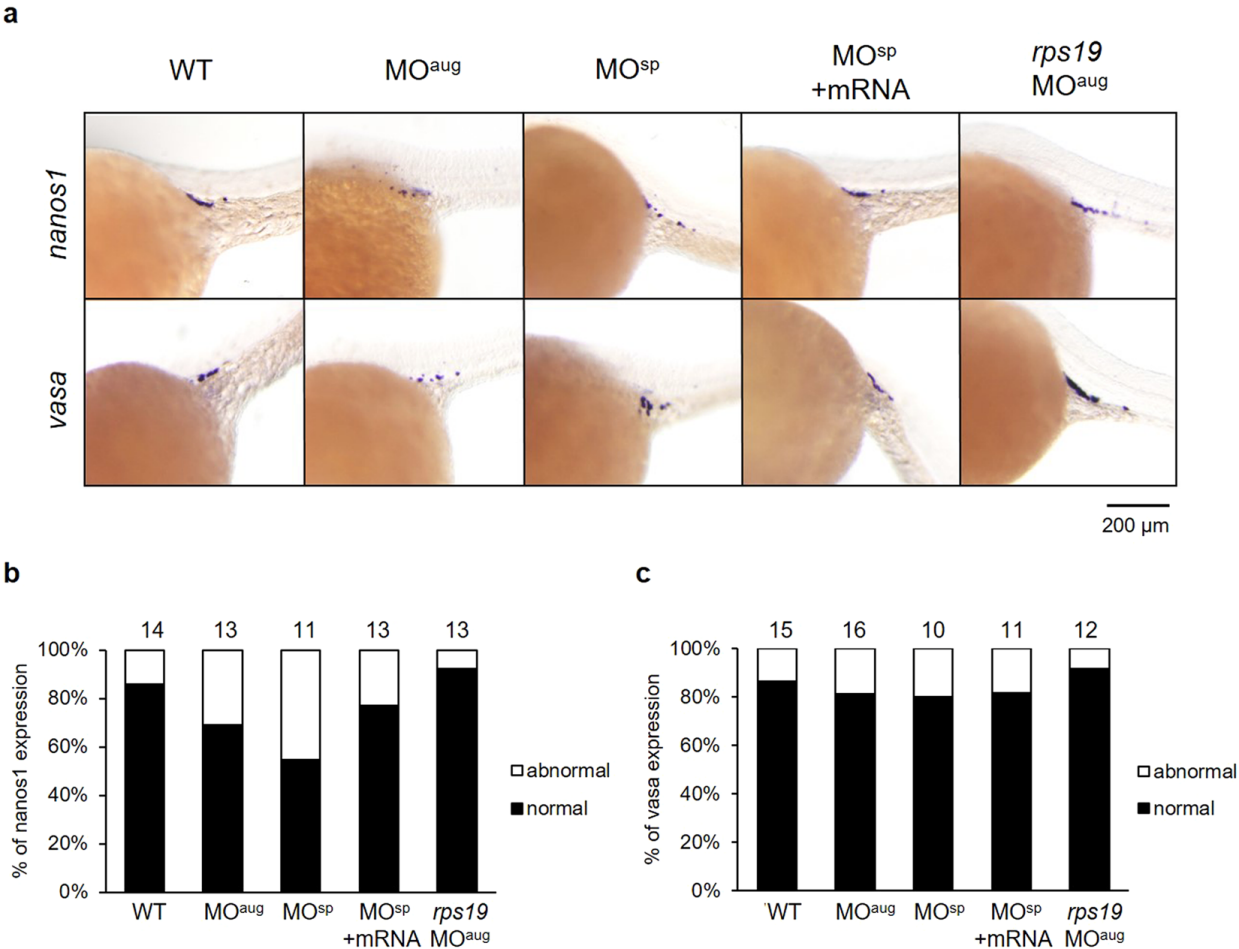
(**a**) Whole-mount *in situ* hybridization showing the expression of PGC marker genes, including *nanos1* and *vasa*, after morpholino injection at 25 hpf. The reduction of *nanos1* and *vasa* expression intensity was obviously observed in MO^aug^-injected embryos. The *nanos1* expression was also decreased in MO^sp^-injected embryos and it was recovered when mRNA was co-injected. The expression levels of *nanos1* (**b**) and *vasa* (**c**) were graded as the normal and abnormal percentage. The mean pixel intensity of *nanos1* and *vasa* gene expression of the normal embryos were 24.7 ± 8.7 and 22.6 ± 8.2, respectively. An abnormal level of the gene expression was taken as one with the value less than the mean minus one standard deviation of the gene expression of the wild-type, i.e., less than 16 for *nanos1* and 14.4 for *vasa* gene. The number of animals quantified in each group are shown on top of the bars.

PGC marker genes in zebrafish embryos were also used to study the effect of *rpl10a* knockdown on the germline using whole-mount *in situ* hybridization. The *vasa* and *nanos1* genes were selected as PGC markers after *rpl10a* gene knockdown at 25 hpf, and *rps19* knockdown morphants were also analyzed as controls. The results showed that after *rpl10a* gene knockdown, the *nanos1* and *vasa* transcripts localized in PGCs were reduced. Although most *rpl10a* morphants exhibited low PGC marker gene expression, some displayed no difference in expression from the wild-type. The *rps19* morphants seemed similar to the wild-type. Although the decreased intensity referred to the reduction of PGC numbers, we did not count the number of PGCs but evaluated the level of intensity instead. The signals from *nanos1* and *vasa* gene expression were divided into 2 groups to estimate the effect of the knockdown. Representative *in situ* hybridization samples are shown in Fig. 8a. The results showed that *nanos1* had lower expression in *rpl10a* MO^aug^- and MO^sp^-injected embryos than it did in the other groups (Fig. 8b). The embryos rescued with *rpl10a* mRNA could recover *nanos1* expression, similar to the wild-type group. There was little difference in *vasa* gene expression in *rpl10a* MO^sp^ and the rescue experiment or control groups (Fig. 8c). These results indicated that *rpl10a* gene knockdown reduced PGC marker gene expression, especially for *nanos1*. Moreover, there was no decrease in PGC marker gene expression in *rps19* MO^aug^ morphants. The whole-mount *in situ* hybridization results also showed the same trends in expression as did the qPCR results.

## Discussion

Haploinsufficiency of RPs caused by loss-of-function mutations in diploid organisms results in typical phenotypic characteristics during development. The first identified RP was the *Drosophila minute* gene, which was reported by Kongsuwan *et al*.^20^ and described by Marygold *et al*.^21^. *Minute* mutants showed developmental retardation, short and thin bristles, irregular eyes, and reduced viability. An RPS6 mutation in *Drosophila* resulted in abnormal blood cell differentiation, slow development, and larval lethality due to the growth inhibition of larval organs^22^. Our results showed that Rpl10a insufficiency showed a severe phenotype and led to a delay in embryonic development in zebrafish. These altered phenotypes might occur because of changing translation or cell cycle arrest stimulation^23^. In addition, swelling of the yolk sac indicated nutrient accumulation and retention in the yolk sac. The nutrient accumulation might suggest that mutant fish have low metabolism. Moreover, the decrease in eosin staining in muscle cells indicated that the muscle has degenerated and resulted in the movement problem in mutant fish.

In addition, using morpholinos to knock down several RPs in zebrafish embryos seems to have more effects than haploinsufficiency does. For example, *rps19* knockdown triggered anemia, which may be caused by apoptosis in the erythropoietic system^24^. Moreover, *rpl11* knockdown directly resulted in head abnormalities and Tp53 induction^12^. The effects of RP knockdown lead to Tp53 buildup, cell death, and developmental retardation^25^. Many ribosomal protein deficiencies lead to inpatient treatment for anemia^2–4,6–8^. The *rpl35*, *rpl35a*, and *rplp2* gene morphants showed severe hemoglobin reductions in blood cells. Similar to *rps19*, gene knockdown affected the erythropoietic system and DBA anemia^24^. Haploinsufficiency of RPs can cause abnormal phenotypes during development, alterations in cell development, lethality, growth inhibition, and anemia^23^. The Rpl10a deficiency might reduce the red blood cells caused by apoptosis of the erythropoietic system, similar to the result of Rps19 deficiency.

Usually, RP mutations in either the small or large ribosomal subunits caused failure in the binding of the 40 S and 60 S subunits. An insufficiency of RPs caused decreases in translation efficiency. The failure of the 40 S or 60 S subunit led to a reduction in protein translation; therefore, the position of erythroid progenitor cells downstream of the protein corresponded to erythroid defects^6,26^. Our findings indicated that Rpl10a-deficient zebrafish had reduced hemoglobin levels but increased the expression of *tp53*. While Torihara *et al*. (2011) indicated that in Rps19 deficiency, the Tp53-independent pathway is more important for the anemia phenotype, the morphological abnormalities were dependent on Tp53 activation^27^. However, the exact role of Tp53, leading to anemia in Rpl10a-deficient zebrafish, is still unknown. Additionally, ribosomal protein deficiency might be a proapoptotic defect due to defects in ribosome assembly. This outcome leads to increased Tp53 activity and the stimulation of a proapoptotic phenotype^6^. Ribosomal protein deficiency led to abnormal morphology and development in zebrafish^23^. Dysfunctions in *rpl10a* gene resulted in morphological abnormalities and growth retardation. Rpl10a-deficient zebrafish died between 3–7 dpf. Early embryonic lethality might be caused by Tp53 activation and apoptosis, although there is no actual evidence for cell death and rescue of cell death upon loss or gain of *rpl10a* function.

In addition, the knockdown of zebrafish *nanos1* by morpholino injection led to fewer PGCs and defects in migration^17^. In our study, we observed that the expression levels of *nanos1* and *vasa* mRNA were significantly reduced in *rpl10a* morpholino-injected embryos. This outcome suggests that PGC reductions and migration defects occurred. The loss of Rpl10a might cause PGC to die due to apoptosis. The expression of *tp53* was upregulated in Rpl10a*-*deficient embryos. On the other hand, *nanos1* and *vasa* gene expression were decreased. Defect of the *rpl10a* gene caused PGC marker genes to decrease, and these genes played an important role in the survival of PGCs. Therefore, the PGC numbers could be reduced. However, the function of *rpl10a* in reproductive organ development requires further study.

In summary, our findings show that *rpl10a* knockdown and knockout zebrafish will be useful models to investigate the additional functions of ribosomal proteins in several pathways and organs in the future.

## Methods

### Animals

Zebrafish (wild-type AB line) were raised and maintained according to standard laboratory conditions in the Bio-resource Division at the Frontier Science Research Center, University of Miyazaki, Japan. The embryos were raised in E3 embryo medium at 28.5 °C. All experimental procedures were performed in accordance with relevant guidelines and regulations and approved by the University of Miyazaki.

### Morpholino injections

Two types of MOs were obtained from Gene Tools, LLC (OR, USA), to knock down the *rpl10a* gene. The AUG MO (MO^aug^: 5′-GACCTTGCTCATTTTGGCGTGATAT-3′) contained 13 bp of the 5′ UTR, the translation start site, 2 bp of exon 1 and 7 bp of exon 2 to block Rpl10a protein translation. Splice MOs were designed to target pre-mRNA splicing at exon 5/intron 5 (MO^sp^: 5′-ATCACAAATATAGACATACCTTCTT-3′). The *rps19* MO^aug^ (5′-CACTGTTACACCACCTGGCATCTTG-3′) was used to develop an anemia phenotype described previously^24^. MOs (MO^aug^ at 0.5 µg/µl; MO^sp^ at 5 µg/µl) were injected into one- or two-cell-stage embryos (wild-type AB line) using an IM-30 Electric Micro-injector (Narishige, Japan). The morphology was observed at 25 and 50 hpf.

### *In vitro* mRNA synthesis for rescue experiments

The full-length coding sequence of *rpl10a* (GenBank accession number NM_199636.1) was amplified using the specific primers shown in Table 1. The purified PCR product and the pCS2^+^ vector (provided by Dr. Kunio Inoue, Kobe University, Japan) were digested with the *Bam*HI and *Eco*RI restriction enzymes (NEB, US), ligated and transformed into DH5α competent *E*. *coli* cells (Takara, Japan). The recombinant *rpl10a*-pCS2^+^ plasmid was linearized using the *Not*I restriction enzyme. Then, mRNA was transcribed using an mMESSAGE mMACHINE SP6 Transcription kit (Ambion, CA, USA). A mixture of MO^sp^ (5 µg/µl) and synthesized *rpl10a* mRNA (400 ng/µl) was used for rescue experiments.

**Table 1:** Primer sequences for RT-PCR and qRT-PCR.

Primer name	Sequence (5′ to 3′)
*ztp53* (F)	CCCATCCTCACAATCATCAC
*ztp53* (R)	TTGCTCTCCTCAGTTTTCCTG
*zrpl10a* (F)	CGTTGTACGAGGCCGTTAAG
*zrpl10a* (R)	AGCTCTGACGTTTTGCCAG
*zrpl38* (F)	ATGCCACGTAAAATCGAAGAA
*zrpl38* (R)	ATCTACTTCAGCTCCTTCACAGC
*zrpl10a*_full (F)	CGGGATCCGCCAAAATGAGCAAGGTCTC
*zrpl10a*_full (R)	GGAATTCTTGTAGAAAACTGAGGAACAGAGTC
*zrpl10a*_HMA (F)	ATGTGCTCAGTGCTGTAGCT
*zrpl10a*_HMA (R)	GTGGATTTCACCTCATCCACCT′
*zgata1* (F)	ATTATTCCACCAGCGTCCAG
*zgata1* (R)	TGGGGTTGTAGGGAGAGTTTAG
*zhbae3* (F)	GCAAAGGACAAAGCGAACGT
*zhbae3* (R)	AGGAGAGTTGGGGCTTAGGT
*zhbbe1* (F)	GCTCTGGCAAGGTGTCTCAT
*zhbbe1* (R)	TTCTTCACTGCCAGCTCCAG
*znanos1_*qPCR (F)	TGCGAGTTTGCATGCATGTG
*znanos1_*qPCR (R)	AACACAACACCAGTGCACAC
*zvasa_*qPCR (F)	AAGGGCTGCAATGTTCTGTG
*zvasa_*qPCR (R)	TGCGCATTTCTGGCTCAAAG
*znanos1*_ISH (F)	GAGCAGCATGGCTTTTTCTC
*znanos1*_ISH (R)	ACACAACACCAGTGCACACA
*zvasa*_ISH (F)	CACTGGGAGAAGAGGCTTTG
*zvasa*_ISH (R)	CAGGTCCCGTATGCAAACTT

### *rpl10a* CRISPR injection

The crRNA was designed to target exon 5 of *rpl10a* gene (GenBank accession number NW_001879347) using the CRISPRdirect website (http://crispr.dbcls.jp/) and synthesized by the Integrated DNA Technology (IDT) company. A mixture of crRNA (25 pg), tracrRNA (100 pg), and Cas9 protein (400 pg) was injected into 115 zebrafish embryos at the 1-cell stage. Five embryos at 24 hpf were taken for the heteroduplex mobility assay (HMA), according to Kotani *et al*.^28^. The primer sequences for the HMA assay are shown in Table 1.

### Semi-quantitative RT-PCR and qRT-PCR

Total RNA was extracted from the wild-type, *rpl10a* MO-injected and homozygous mutants using TRIzol reagent (Invitrogen, CA, USA). The samples were randomly collected at 24 and 48 hpf. cDNA synthesis was performed using a High Capacity cDNA Transcriptase kit (ABI, CA, USA). Semi-quantitative analysis of *rpl10a* and *rpl38* was carried out using an Expand™ High Fidelity PCR System kit (Roche, Mannheim, Germany). The quantitative real-time PCR analysis was carried out to assess apoptosis-, erythropoiesis-, and germ cell-related mRNAs using 5 ng of cDNA as a template, specific primers at a concentration of 300 nM, and 1x FastStart SYBR Green Master Mix (ABI, CA, USA). The fold changes in *tp53*, *gata1*, *hbae3*, *hbbe1*, *nanos1* and *vasa* gene expression were determined using the 2^−ΔΔCT^ method. Gene expression was normalized to that of the *rpl38* gene. The primer sequences for PCR are shown in Table 1.

### Histology

The embryos at 3 dpf were selected for histological analysis. The tails of the embryos were cut from individuals in the fish water with tricaine to confirm their genotypes. Then, the embryos were fixed in 4% paraformaldehyde in PBS for 24 h and then washed with PBS. After rinsing, the embryos were placed in a tissue processor and embedded in paraffin blocks. The paraffin blocks were sectioned using a rotary microtome and placed on a slide. The sections were deparaffinized using toluene, rehydrated with an ethanol gradient and stained with hematoxylin and eosin. The morphology was observed using an Olympus BX53 microscope (Olympus, Tokyo, Japan).

### Hemoglobin staining

Embryos at 48 hpf were stained using *o*-dianisidine to detect active hemoglobin^29^. Dechorionated embryos were incubated with staining buffer containing 0.6 mg/mL *o-*dianisidine, 0.01 M sodium acetate pH 4.5, 0.65% H_2_O_2_ and 40% ethanol for 7 min in the dark.

### Whole mount *in situ* hybridization

Digoxigenin-labeled RNA probes were synthesized against *vasa* and *nanos1* mRNA. Each PCR product was amplified from wild-type zebrafish cDNA pool using specific primers shown in Table 1. The PCR fragments were purified using a MagExtractor PCR & Gel Clean-up kit (Toyobo, Japan) and ligated into the pGEM^®^-T Easy vector (Promega, WI, USA). The ligation mixture plasmid was transformed into DH5α competent *E*. *coli* cells. *In vitro* transcription was performed using a T7 or SP6 RNA polymerase kit (Roche, Mannheim, Germany) with linearized plasmid as a template. The zebrafish embryos at 24 hpf were dechorionated and fixed in 4% (w/v) paraformaldehyde (PFA) in 1x PBS overnight at 4 °C. The embryos were dehydrated with 50% methanol in PBS and in 100% methanol. Subsequently, the embryos were rehydrated in 75, 50, and 25% (v/v) methanol in PBS and washed with PBST (1x PBS pH 7.4 + 0.1% Tween 20) for 5 min each. Then, the samples were permeabilized by incubating with 10 µg/mL proteinase K for 10 min. The embryos were soaked in the hybridization buffer for 2 h at 56 °C for prehybridization, and incubated with each RNA probe (30–50 ng) for 18 h. The embryos were rinsed with 2x SSCT in 50% formaldehyde for 1 h at 56 °C. The RNase treatment (RNase A and RNase T1) step was performed over 10 min to remove endogenous RNA. The solution was removed and incubated with 2x SSCT in 50% formaldehyde for 1 h at 56 °C, followed by incubation with 2x SSCT, 0.2x SSCT, and PBS. The nonspecific binding sites were saturated by soaking in blocking buffer at 4 °C for 2 h. Afterward, the embryos were incubated with anti-DIG antibody (1:3,000) in blocking buffer overnight at 4 °C with gentle shaking. On the last day, the samples were washed with PBST and alkaline reaction buffer. An NBT/BCIP substrate was added to detect alkaline phosphatase until the color developed, and the reaction was stopped by rinsing with PBST.

The expression levels of the PGC marker genes were roughly graded into two groups, including abnormal and normal expression. The expression level of the *nanos1* and *vasa* was measured using Fiji analysis software (https://fiji.sc). The image of individual embryos was captured under microscopy. Then, the images were inverted and converted to 8-bit grayscale. A region of interest (ROI) was manually outlined and measured around expression signal at yolk sac extension area of each embryo. Then the ROI with the same shape and the same area was moved to the region that contained no signal. This intensity was determined as a background. The intensity value of the background region was subtracted from the stained region. The intensity of ISH signal was calculated as pixel intensity individually. Due to the variability of the expression signal, the level of intensity was scored as normal and abnormal. An Abnormal level of the gene expression was taken as one with the value less than the mean minus one standard deviation of the gene expression of the wild-type. While the normal level was the mean pixel intensity of the gene expression of the wild-type ± SD. The percentage of normal and abnormal expression of the *nanos1* and *vasa* gene of each group were then determined.
